# Quantification of the impact of aerosol on broadband solar radiation in North China

**DOI:** 10.1038/srep44851

**Published:** 2017-03-21

**Authors:** Bo Hu, Xiujuan Zhao, Hui Liu, Zirui Liu, Tao Song, Yuesi Wang, Liqin Tang, Xiangao Xia, Guiqian Tang, Dongsheng Ji, Tianxue Wen, Lili Wang, Yang Sun, Jinyuan Xin

**Affiliations:** 1 State Key Laboratory of Atmospheric Boundary Layer Physics and Atmospheric Chemistry (LAPC), Institute of Atmospheric Physics, Chinese Academy of Sciences, Beijing 100029, China; 2 Institute of Urban Meteorology, Chinese Meteorological Administration, Beijing 100089, China; 3 College of Atmospheric Sciences, Lanzhou University, Lanzhou 730000, China; 4 Sub-center of atmospheric science of Chinese ecosystem research network, Beijing 100029, China; 5 LAGEO, Institute of Atmospheric Physics, Chinese Academy of Sciences, Beijing, China

## Abstract

PM2.5 plays a key role in the solar radiation budget and air quality assessments, but observations and historical data are relatively rare for Beijing. Based on the synchronous monitoring of PM2.5 and broadband solar radiation (*R*_s_), a logarithmic function was developed to describe the quantitative relationship between these parameters. This empirical parameterization was employed to calculate *R*_sn_ from PM2.5 with normalized mean bias (NMB) −0.09 and calculate PM2.5 concentration from *R*_sn_ with NMB −0.12. Our results indicate that this parameterization provides an efficient and straightforward method for estimating PM2.5 from *R*_s_ or *R*_s_ from PM2.5.

Aerosol particles not only impair the direction of solar radiation through the extinction effect but also interfere with the progress of atmospheric longwave radiation, which influences the energy balance^1^. The direct and indirect effects of aerosols on *R*_s_ in the megalopolis of Beijing and its surrounding areas have been discussed by many scholars and researchers. For example, *R*_s_ in Beijing has declined at a rate of 5% per decade since the late 1980s, and this pattern is similar to the decrease of cloud cover over the same period. Previous studies noted that the aerosol concentration may play a more important role in *R*_s_ dimming than cloud cover^2^,^3^,^4^. They also suggested that enhanced absorption by the aerosol particles can explain why the diffuse irradiance in the Beijing area has decreased more than 3% per decade, accompanied by an increase in aerosol loading over the last 40 years.

Accompanied by accelerated urbanization and industrialization, remarkably poor air quality and haze pollution have appeared in most regions of China^5^,^6^. Regionally severe and complicated air pollution (coal smoke pollution, photochemical smog, etc.) has frequently appeared and is even worse in city clusters. For example, the annual average concentration of the aerosol particle PM2.5 (airborne particles with aerodynamic diameters less than 2.5 μm) reached 72.3 μg m^−3^ in Beijing from 2004 to 2012. This concentration exceeded 2–5 times that in other developed countries^5^,^7^. It is well known that aerosols lead to a significant impairment in the radiation balance due to the scattering and absorption effects on radiant transfer through the atmosphere. Aerosols also weaken the turbulence in the atmosphere, which restrains the development process of the planetary boundary layer (PBL), followed by a weakening of the atmospheric pollutant dispersion ability, leading to heavy pollution^8^,^9^. There is no doubt that aerosols play an important role in both global energy balance and the environment. However, quantitative evaluations of aerosol effects on the environment are problematic, especially in the PBL, where evaluation is very difficult due to the large uncertainties in the radiative properties of atmospheric aerosols, which are determined by the chemical and physical properties of aerosol. It should be noted that studies that have focused on the effect of aerosols on solar radiation variation, and PBL evolution processes are mainly based on hypotheses.

Many researchers have sought to retrieve the PM2.5 concentration from the aerosol optical depth (AOD), such as in the U.S. Environmental Protection Agency’s AIRNow program^10^,^11^,^12^. As we know, PM2.5 concentration and AOD are two significantly different physical quantities. The PM2.5 concentrations represent aerosol particle loading at the surface, whereas AOD characterizes the instantaneous integrated vertical profiles of the aerosol extinction coefficient. Fortunately, more than 80% of the aerosol particles are agglomerated in the surface layer, which indicates that the relationship between the PM2.5 concentration and AOD can be quantitatively measured. Therefore, a widely used empirical equation has been established to convey the relation between AOD and the PM2.5 concentration. For example, Al-Saadi *et al*.^12^ revealed that AOD can be used to reconstruct the PM2.5 concentration, with the conversion factor of AOD to PM2.5 concentration ranging from 25 to 60 (mg/m^3^/AOD) over the eastern U.S., and indicated there are more physical links between AOD and PM2.5 concentration under the appropriate assumptions and created theoretical and reliable methods to convert AOD to PM2.5 concentration^13^.

Xia^14^ provided a straightforward method to estimate *R*_s_ under clear sky conditions with AOD and indicated that AOD can be directly calculated from *R*_s_ and other parameters. Li *et al*.^15^ reported that the PM2.5 concentration could be accurately estimated from the fine-mode aerosol optical depth, and Tsai *et al*.^16^ evaluated the accuracy of estimating the PM2.5 concentration from AOD and confirmed that AOD could be used to obtain acceptable PM2.5 concentrations.

Similar to the AIRNow program methods, the relationship between *R*_s_ and the PM2.5 concentration is investigated in this study. An empirical equation is established to convert *R*_s_ to the PM2.5 concentration. Long-term simultaneous *in situ* measurements of PM2.5 concentrations and solar radiation in Beijing provide a chance to quantitatively analyse the interaction between PM2.5 concentration and *R*_s_.

The aim of this study was to explore the variation in the temporal properties of PM2.5 and *R*_s_ from sunrise to sunset using *in situ* data from 2005 to 2015 in Beijing. In addition, a straightforward equation for estimating PM2.5 from *R*_s_ was developed based on the quantitative relationship between PM2.5 and *R*_s_.

## Results

**Figure 1:**
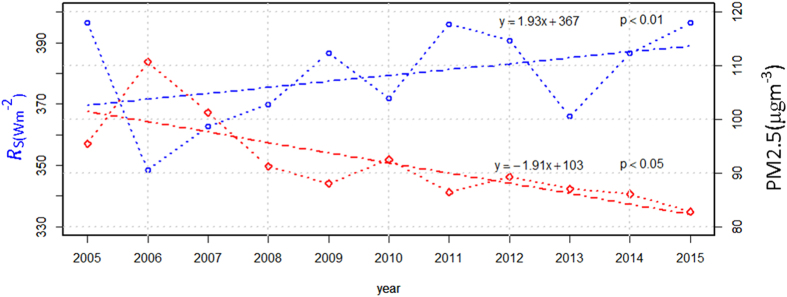
Variation of the PM2.5 concentration and *R*_*s*_ in Beijing from 2005 to 2015. P < 0.01 denotes that this linear trend is statistically significant at the 99% confidence level. P < 0.05 denotes that this linear trend is statistically significant at the 95% confidence level. The figure was produced using MATLAB.

The annual variation in the PM2.5 concentration over Beijing during the study period is presented in Fig. 1. The average annual value for 2005–2015 was 91.9 μg m^−3^. The highest annual average occurred in 2006, and this was caused by increased occurrences of dust events in the spring of 2006. The PM2.5 concentration during the study period was evidently lower than that observed in 2000 in Beijing^17^, where the values reached 127 μg m^−3 ^. However, the annual PM2.5 concentration in Beijing during the study period was 6 times higher than the National Ambient Air Quality Standard of the U.S. The PM2.5 concentration in Beijing has persistently declined since 2006, with a rate of decline of 1.91 μg m^−3^ per year, and this decreasing trend was statistically significant at the 95% confidence level. The PM2.5 concentration in Beijing has remained at a relatively low level since 2008, and the trend has decreased sharply over the past 3 years. These results indicate that the implementation of the recent air pollution improvement programme in Beijing has reduced fine particle pollution.

The average value of *R*_*s*_ during 2005–2015 was 379.2 W m^−2^. The lowest annual average occurred in 2006 and was due to the highest PM2.5 concentration that also occurred in 2006. The *R*_*s*_ in Beijing has been increasing since 2006, with a rate of increase of 1.93 W m^−2^ per year, and this increasing trend was statistically significant at the 99% confidence level. At the same time, there were significant decreasing trends of PM2.5 concentration in Beijing during this period. The trend variations of *R*_*s*_ were opposite to the trends of PM2.5.

**Figure 2:**
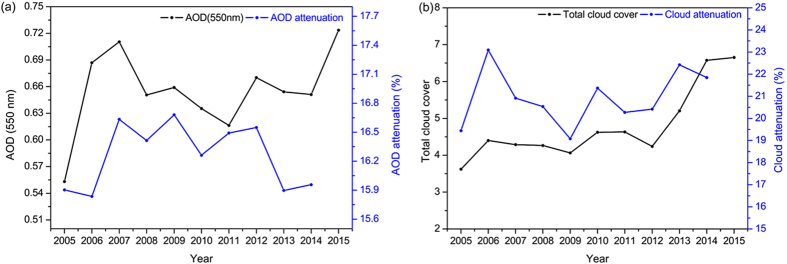
Annual variations of the AOD, cloud cover and the extinction effects of the AOD and cloud cover relative to *R*_*s*_ attenuation. (**a**) Time series of AOD and the contributions of the AOD to *R*_*s*_ attenuation; and (**b**) time series of cloud cover and the contributions of the cloud cover to *R*_*s*_ attenuation. Cloud cover obtained from CMA. The figure was produced using OriginPro.

As mentioned above, the amount of *R*_*s*_ received at the Earth’s surface mainly depends on the extinction effects caused by aerosols and clouds. The trends of AOD and the attenuation of *R*_*s*_ by AOD are shown in Fig. 2. As shown in this figure, the trends of AOD are not precisely consistent with those of PM2.5, and this difference can be explained by the fact that AOD is calculated for clear sky conditions rather than PM2.5, which is calculated for all sky conditions. The trends of AOD or AOD attenuation are not in concert with *R*_*s*_ and cannot completely explain the increase of *R*_*s*_ in Beijing. This may be due to the AOD values only being available under clear sky conditions. Of course, the other main influence of cloud cover on *R*_*s*_ should be considered when explaining the variation characteristics of *R*_*s*_. Figure 2b depicts the changes in cloud cover and the attenuation effect of cloud cover on *R*_*s*_ during this period. The surface observed cloud cover is provided by the Meteorological Information Comprehensive Analysis and Process System (MICAPS) from 2005 to 2015. There was a negative correlation between *R*_*s*_ and the attenuation effect of cloud cover on *R*_*s*_. The total cloud cover showed a weak increase trend before 2012 and clearly increased after that. The attenuation effect of cloud cover on *R*_*s*_ generally increased during the study period and could not explain the increase of *R*_*s*_. Thus, the increase trend of *R*_*s*_ is most likely caused by the decrease of aerosol concentration. In this study, we try to prove this assumption with analysis of the quantitative relationship between PM2.5 concentration and *R*_*s*_.

Previous research has noted that an accurate *R*_*s*_ can be obtained under clear conditions using SBDART^4^ and parameterization methods^14^. The surface albedo, ozone, water vapour, AOD, single-scattering albedo and asymmetry factor were the essential parameters used by SBDART to model *R*_*s*_. The ozone data were downloaded from OMI (http://acd-ext.gsfc.nasa.gov/Data_services/merged/index.html), and water vapour data were obtained from AERONET. The input parameters of aerosol were also obtained from AERONET. We interpolated and extrapolated the SBDART method to calculate the aerosols’ parameters to meet the spectral signatures of the SBDART model. We used the data collected in 2013 in Beijing to evaluate the performance of SBDART.

**Figure 3:**
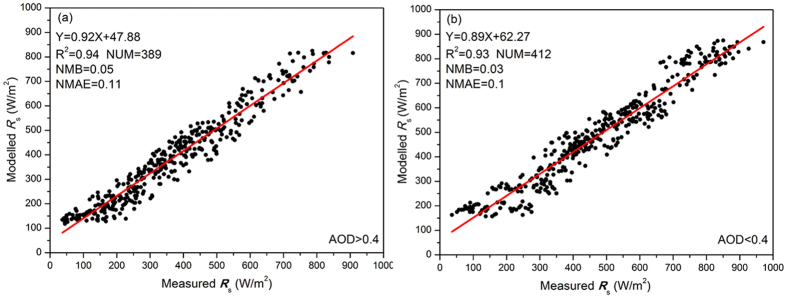
Comparison of measured and modelled *R*_*s*_ based on measured AOD under clear and haze-fog events in 2013 in Beijing. The figure was produced using OriginPro.

AOD (440 nm) was less than 0.38 under clear sky conditions and greater than 0.4 in haze-fog weather. We used this categorization to classify clear and haze-fog conditions. Figure 3 compares the measured and modelled values of *R*_*s*_. The model simulations can account for more than 94% of the measurement variances in both clear and haze-fog conditions.

The statistical parameter normalized mean bias (NMB) and normalized mean absolute error (NMAE)^18^ were used to evaluate the performance of the model. The NMB values between the simulations and measurements were 0.03 and 0.05 in clear and haze-fog conditions, respectively. The NMAE were less than 0.1 under both sky conditions. And this indicates the model overestimates the observations by a factor of 1.05, the absolute gross error is 1.1 times the mean observation and model prediction for over prediction. The linear fits show statistically significant values at a 99% confidence level. The statistical results show that under both clear and haze-fog conditions, SBDART can produce a satisfactory result, which indicates that *R*_*s*_ can be accurately calculated from AOD using this radiative transfer model. As mentioned before, AOD can be inversed from PM2.5 concentration, and this indicated that the parameters of PM2.5 concentration can be used to calculate *R*_*s*_ with an acceptable accuracy. In other words, the relationship between PM2.5 concentration and *R*_*s*_ can be used to reconstruct one from the other.

**Figure 4:**
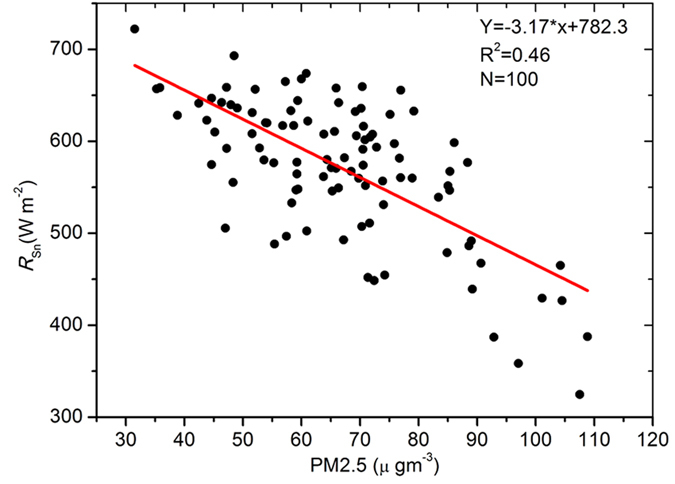
Comparison of the monthly average of PM2.5 concentration to *R*_*sn*_ measurements in Beijing from 2005 to 2015. The figure was produced using OriginPro.

**Figure 5:**
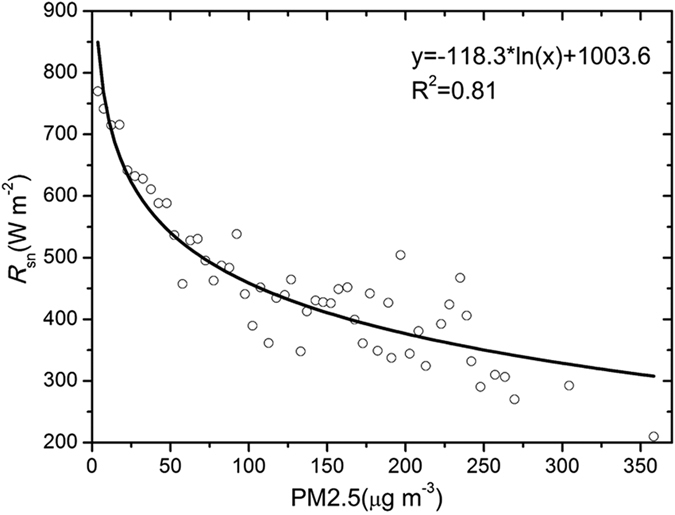
Scatter plot of the dependence of *R*_*sn*_ on PM2.5 concentration using measured data from 2005 to 2010. The figure was produced using OriginPro.

The monthly averages of PM2.5 concentration and *R*_*sn*_ were used to analyse the influence of PM2.5 on *R*_*sn*_. In Fig. 4, we can see that there is a good negative linear correlation between the monthly averages of *R*_*sn*_ and PM2.5 concentration in Beijing. This result indicates that one of these two parameters can be estimated from each other using the empirical equation. The amount of *R*_*sn*_ primarily depends on the solar zenith angle (*θ*) and extinction effects by gases and particles; thus, we first dispelled the influence of the zenith angle on *R*_*sn*_ by multiplying the sine of the solar zenith angle, referred to as the normalized *R*_*sn*_. Then, we classified the measured PM2.5 into different bins and calculated the average *R*_*sn*_ for each bin. The scatter plot of the dependence of *R*_*sn*_ on PM2.5 from 2005 to 2010 is presented in Fig. 5. There is a logarithmic relationship between PM2.5 and *R*_*sn*_. The parameterization equation for calculating *R*_*s*_ from PM2.5 is as follows:


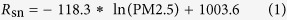



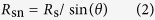


where *R*_*s*_ is *in situ* measured data, *θ* is the solar zenith angle, and PM2.5 concentration is the measured concentration of PM2.5.

**Figure 6:**
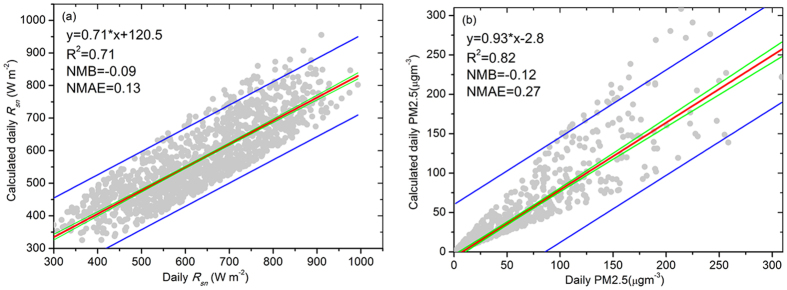
Scatter plot of the *in situ* measured and estimated values using the proposed method using data measured in Beijing from 2011 to 2015. (**a**) Comparison of measured and modelled *R*_*sn*_; and (**b**) comparison of measured and modelled PM2.5 concentrations. The fitted regression line (in red), the 90% confidence limits (in blue), and the 95% prediction limits (in green) are displayed. The figure was produced using OriginPro.

To evaluate the effectiveness of the empirical parameterization for *R*_*sn*_ from the measured PM2.5 concentration or the aerosol concentrations estimated from measured *R*_*sn*_, measured daily *R*_*sn*_ and PM2.5 concentration from 2011 to 2015 were used. The comparison results show that there was a good linear relationship between the measured and modelled values, except for 1% out of the 90% confidence level limits (Fig. 6). The slope of the linear regression was less than 1, which indicated that this method underestimates *R*_*sn*_. The NMB and NMAE were −0.09 and 0.13, respectively. The performance of the method in calculating the PM2.5 concentration is shown in Fig. 6b. The slope was near 1, and the NMB and NMAE were −0.12 and 0.27, respectively. When we compared this result to the relative error between the measured and inversely calculated PM2.5 concentration from AOD in Beijing^19^, this method provides more reliable results for further analysis. This indicates that the parameterization equation established in this study can provide reliable inversion results for both *R*_*sn*_ and the PM2.5 concentrations.

The *in situ* measured data from Xianghe and Shangdianzi stations were used to test the transferability of this estimation method. The statistical results of linear regression between calculated and observed data indicated that this method can provide an acceptable calculated PM2.5 concentration and *R*_*sn*_ for the NCP region (Figs S2 and S3).

**Figure 7:**
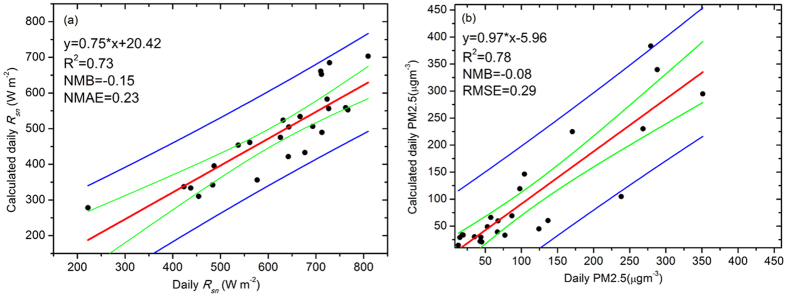
Comparison of the estimated and measured values of (**a**) *R*_*sn*_ and (**b**) PM2.5. The fitted regression line (in red), 90% confidence limits (in blue), and 95% prediction limits (in green) are displayed. The figure was produced using OriginPro.

To assess the performance of the method in extreme pollution events, the heavy haze pollution episode in January 2013 in Beijing was used to evaluate the estimation equation. Figure 7 shows the comparison between measured *R*_*sn*_ values and calculations from the parameterization Eq. (1). The NMB and NMAE values were −0.15 and 0.23, respectively. The slopes of both the measured and modelled *R*_*sn*_ and PM2.5 slightly improved, especially the inversion results of PM2.5, whose slope and NMB were 0.97 and −0.08, respectively. These results again indicate that this parameterization method provides reliable results in any event, besides the model underestimates the observations by a factor of about 1.13, the absolute gross error is 1.23 times the mean observation and model prediction for over prediction.

As we know, aerosol can be directly and indirectly influence energy balance via scattering and absorbing effects, and then impact climate. Guo *et al*.^20^ and Zhang *et al*.^21^ claimed that there is a significant periodic cycle of aerosol concentration in Beijing, and this cycle is mainly controlled by the meteorologic condition. The periodic cycle of PM2.5 concentration presented as a 2- to 7-day variation cycle. The *in situ* measured data from the January 2013 heavy haze pollution episode has been used to investigate variation trends of *R*_*sn*_ and PM2.5 concentration in Beijing. There is a significant opposite change trend of *R*_*sn*_ compared with PM2.5 concentration (Fig. S4).

**Figure 8:**
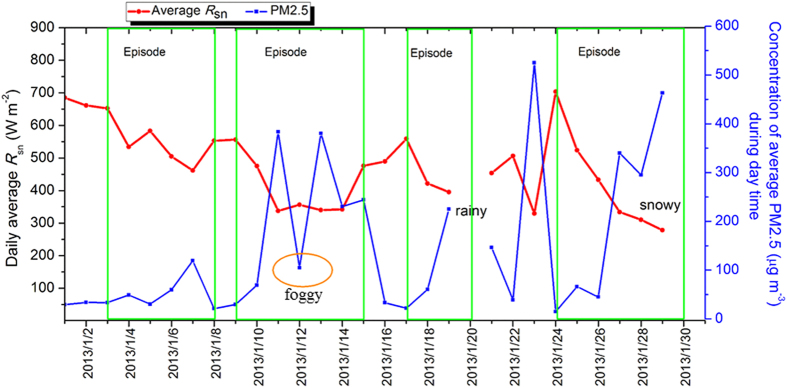
Periodic variation cycles of calculated *R*_sn_ and PM2.5 concentration in Beijing during the heavy haze pollution episode of January 2013. The figure was produced using OriginPro.

The variation of calculated PM2.5 concentration and *R*_sn_ is presented in Fig. 8, and the missing data was due to rainy and snowy weather processes. The calculated PM2.5 concentration and *R*_sn_ were not consistent with the *in situ* measurements on January 12, 2013, which may be caused by the dense fog. Evidently, the variation cycle of the calculated *R*_*sn*_ and PM2.5 concentration during the January 2013 pollution period exhibits a clear variation cycle, the same as the measured results.

## Discussion

A series of reports indicated that the impact of aerosols on downwelling solar irradiance (*R*_s_) was significant and simultaneously impaired visibility in most parts of China^2^,^3^,^4^. Studies of sky dimming or brightening across the whole world have focused on long-term variations of *R*_s_. The variation of *R*_s_ in China was significantly different from other regions, which mainly presents the decrease of *R*_s_ along with cloud cover decreasing. This indicated that dimming or brightening could not be explained solely by the variation in cloud cover. Many previous results recognized that a sharp increase in anthropogenic aerosol was sufficient to explain the dimming accompanied by cloud cover decrease in China. Liang and Xia^22^ provide a reliable explanation for the transition of *R*_s_ from a tendency to decrease to no significant trend since the 1980s, due to the increments of the aerosol single scattering albedo during this period. According to the analysis in this study, it can be concluded that the long-term variation of PM2.5 concentration has a significant influence on the tendency of *R*_s_ in China, especially in city clusters, and this can be used to estimate the PM2.5 concentration and other parameters, which is very important for air quality studies, especially in the areas outside cities without routine measured PM2.5 concentration. Furthermore, this method can prove historic PM2.5 concentration datasets to address the reasons for variation trends of *R*_s_ in China.

In this study, we developed a simple and effective parameterization estimation model for estimating the PM2.5 concentration from the more routine measured *R*_s,_ or *R*_s_ from PM2.5 concentration. The advantage of this method is that the input parameter is simple and more easily obtained, and this will afford a powerful tool for the study of air pollution and interactions between aerosol and radiation. However, this method is not suitable for rain, dust storms, and snow events, and the calculation accuracy decreases when transferred to another site. This may be due to the differences in aerosol size distribution, chemical composition, BC concentration and mixing state of BC. Peng *et al*.^23^ suggested that aged black carbon (BC) aerosol, enhanced by a factor of approximately 2.4 to fresh BC, and BC direct radiative forcing (DRF) are approximately 0.77 W m^−2^. The ageing period is shorter than other sites. These results indicate that the impact of BC on air quality and climate should receive more attention in this region. As we know, the DRF caused by an increased concentration of BC is mainly dependent on the externally or internally mixing state, and atmospheric ageing progress. However, the DRF of BC was still highly uncertain. Therefore, we used the SBDART and aerosol absorption optical depth (AAOD) from AERONET during 2005–2014 to calculate the influence of BC on the energy balance through Beijing (Supplementary Material: The impact of BC on *R*_*s*_ reach at the surface accounted for 21.8% of aerosol on *R*_*s*_ under clear sky conditions (Fig. S5), and this indicated that BC plays an important role in the attention effect on *R*_*s*_. Therefore, we will endeavour to improve the accuracy and transferability of this estimation method on a larger scale, considering the influence of clouds, aerosol physical and chemical properties, and BC on *R*_s_ in further studies.

Aerosol is the main factor impairing visibility and can damage vegetation; at worst, it can cause respiratory difficulties, a variety of other health problems or even premature death. Comprehensive monitoring and analysis of PM2.5 results in societal and economic benefits by advanced planning; therefore, the accurate measurement or estimation of PM2.5 is important. The PM2.5 networks belonging to the China Environmental Protection Agency are still spatially scarce, with only 74 cities measuring PM2.5 in 2013 and 338 cities in 2015. However, these observation stations mainly focus on the city; therefore, the satellite-inverted method has been developed to estimate PM2.5 concentration. Even if acceptable, AOD has been inverted from few satellite observations since 2000 (e.g. the Moderate Resolution Imaging Spectroradiometer (MODIS). The parameterizations in this study provide a straightforward method to calculate PM2.5 from *R*_s_.

In this study, we used global solar radiation measurements that are easier to obtain than direct solar radiation. There were more than 100 stations belonging to the China Meteorological Administration (CMA) and 44 stations belonging to the Chinese Ecosystem Research Network (CERN) that take global solar radiation measurements routinely. The measured *R*_s_ data that came from CMA can be traced back to 1961. Furthermore, many results indicate that sunshine duration can be used to calculate acceptable *R*_s_^24^,^25^ therefore, the highest spatial and temporal distribution of PM2.5 concentration can be obtained by using this method.

## Conclusion

Solar radiation and aerosol are the most important physical quantities that influence climate change and the atmospheric environment. Based on the analysis of the relationship between long-term measured *R*_s_ and PM2.5 concentration, it can be concluded that the long-term variation of PM2.5 has a significant influence on the tendency of *R*_s_ in China, especially in city clusters. An effective parameterization of *R*_s_ using a logarithmic function of these two parameters was developed. Thus, acceptable PM2.5 concentrations could be estimated from *R*_s_, which significantly improved PM2.5 revision from AOD. Another advantage was that this straightforward method has been established to estimate *R*_s_ from PM2.5 and vice versa.

## Methods

### Site description

The super aerosol observation station, focused on the physicochemical characteristics of atmospheric particles, was located in downtown Beijing (39°56|N, 116°17|E, and 75.0 m a.s.l.). The station was near the Institute of Atmospheric Physics, Chinese Academy of Sciences (IAP) 325-m meteorological tower, which was located between the Third and Fourth Ring Roads. This location was surrounded by domestic dwellings, Madian Park in the southeast and Yuandadu Park to the east and west. Liu *et al*.^7^ confirmed that this station can be used to represent the atmospheric pollution in Beijing, and Hu *et al*.^26^ declared that this site could feature the energy balance properties in Beijing. The measured results obtained from this station can be used to characterize the average properties of the atmospheric environment in urban Beijing. The suburban station Xianghe (39°47′N, 116°57′E, 95 m a.s.l.) and rural station Shangdianzi (40°39′N, 117°07′E, 293.9 m a.s.l.) are used to evaluate the performance of transferability of the quantitative relationship between *R*_s_ and PM_2.5_ concentrations (Fig. S1).

The Santa Barbara DISORT Atmospheric Radiative Transfer (SBDART) model was used to calculate *R*_s_. The inputs for this model were easy to obtain, such as aerosol optical property parameters, surface albedo and cloud property parameters^27^.

The amount of *R*_*s*_ depends primarily on the solar zenith angle (*θ*) and extinction effects by gases and particles. Quantitative analysis of the relationship between *R*_*s*_ and aerosol loading under clear sky conditions has been performed by Xia^14^ using measured aerosol optical depth and *R*_*s*_, but observations in all sky conditions are limited.

Thus, we used simultaneous observation data of *R*_*s*_ and the PM2.5 concentration during the sunrise to sunset period under all sky conditions, except for rain, dust storms, and snow events, to establish the relationship between these two parameters. First, we dispelled the influence of the zenith angle on *R*_*s*_ by multiplying by sec (*θ*), referred to as the normalized *R*_*s*_ (*R*_*sn*_); second, the loading of aerosol during the day was calculated using the hourly dataset (after excluding rain, dust storms, and snow events); third, in order to reduce the dispersion property of scatter plots between *R*_sn_ and PM2.5, the measured PM2.5 concentration was classified into different bins, starting at 5 μg m^−3^ with a step length of 5 μg m^−3^, and the average *R*_*sn*_ was calculated for each bin. Daily average *R*_sn_ and PM2.5 concentration from 2005–2010 at Beijing station were used to investigate the quantitative relation between these two variables.

Then, the data from 2011 to 2015 at Beijing station and *R*_sn_ and PM2.5 concentration during 2013 and 2014 at Xianghe and Shangdianzi stations were used to evaluate the accuracy of this method.

## Additional Information

**How to cite this article:** Hu, B. *et al*. Quantification of the impact of aerosol on broadband solar radiation in North China. *Sci. Rep.* **7**, 44851; doi: 10.1038/srep44851 (2017).

**Publisher's note:** Springer Nature remains neutral with regard to jurisdictional claims in published maps and institutional affiliations.

## Supplementary Material

Supplementary Information
