# Supplementary material for: Quantification of the impact of aerosol on broadband solar radiation in North China
Source: Sci Rep. 2017 Mar 21;7:44851. doi: 10.1038/srep44851 (PMC5359593; doi:10.1038/srep44851)
Supplement: Supplementary Information [file srep44851-s1.pdf]

Quantification of the impact of aerosol on broadband solar radiation in North China

Bo Hu [1], Xiujuan Zhao[2], Hui Liu [1,3], Zirui Liu[1], Tao Song[1], Yuesi Wang [1,4], Liqin Tang[1], Xiangao Xia [5], Guiqian Tang[1], Dongsheng Ji[1], Tianxue Wen[1], Lili Wang[1], Yang Sun[1], Jinyun Xin[1]

[1]State Key Laboratory of Atmospheric Boundary Layer Physics and Atmospheric Chemistry (LAPC), Institute of Atmospheric Physics, Chinese Academy of Sciences, Beijing 100029, China.

[2]Institute of Urban Meteorology, Chinese Meteorological Administration, Beijing 100089, China

[3]College of Atmospheric Sciences, Lanzhou University, Lanzhou 730000, China.

[4]Sub-center of atmospheric science of Chinese ecosystem research network, Beijing 100029, China.

[5]LAGEO, Institute of Atmospheric Physics, Chinese Academy of Sciences, Beijing, China.

Correspondence and requests for materials should be addressed to B. H (email: hub@post.iap.ac.cn) and Y.S. (wys@dq.cern.ac.cn)

Supplementary data

Site

The simultaneously *in situ* measured R_s and concentrations of $PM_{2.5}$ in Beijing (39°56ˈN, 116°17ˈE, 75.0), suburban station Xianghe (39°47ˈN, 116°57ˈE, 95 m a.s.l.), and rural station Shangdianzi (40˚39′N, 117˚07′E, 293.9 m a.s.l.) are used to develop and evaluate the performance of the quantitative relationship between R_s and concentrations of $PM_{2.5}$. Xianghe station is located in a suburban region in Hebei Province, China, which is located approximately 80 km southeast of central Beijing. Shangdianzi station is located in the rural area Miyun district, and this is one of the regional Global Atmosphere Watch (GAW) stations in China (Fig S1). The measurement instruments of R_s and PM2.5 concentration in Xinaghe and Shandianzi are same as those in Beijing station.

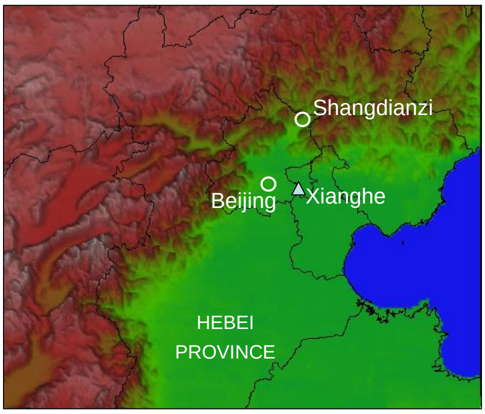

Fig S1 the map of observation sites in the North China Plain (NCP). The map was drawn with Vis5D V5.2 (http://www.ssec.wisc.edu/~billh/vis5d.html) and USGS DEM data （downloaded from ftp://edcftp. cr.usgs. gov/ pub/ data/ gtopo30/ global in http://edc.usgs.gov web）and modified using PowerPoint by Xiujuan Zhao.

Instruments and data quality control

The concentrations of $PM_{2.5}$ were measured by a tapered element oscillating microbalance (Thermo Electron Corporation, TEOM 1400a). This instrument was designated by the US Environmental Protection Agency (USEPA) based on the equivalent method, and this method has been considered as a National Ambient Air Quality standard monitoring compliance used for the real-time monitoring of aerosol concentrations. The temporal resolution of PM2.5 concentration was 5 min. These initial data were used to calculate the hourly average values of PM2.5, and then daily concentrations were obtained using the hourly data set.

The real-time values of R_s (305–2800 nm) were measured by CM-11 pyranometers (Kipp & Zonen, Delft, Netherlands) with an accuracy of 2-3%. The R_s values were recorded at 1-min intervals, and then hourly and daily values were calculated by integrating the real-time values. The CM-11 pyranometers were calibrated using the 'alternate method' (Bruce, 1996). The maximum sensitivity deviations in the CM-11 pyranometers averaged under 0.5% after the calibration process.

The measured results of R_s should be limited to extraterrestrial global solar radiation (R_e), and the smallest acceptable value of the ratio of R_s to R_e was 0.03 (Geiger et al., 2002). The meteorological parameters, such as air temperature, relative humidity, pressure, wind speed, wind direction and rainfall, were recorded by an automatic meteorological monitoring instrument, Milos520 (Vaisala, Finland). All measurement instruments were calibrated and cross-evaluated at the start and end of data collection.

The quality control of concentrations of $PM_{2.5}$ is based on the China environmental protection standard HJ 618-2011. Approximately 7.5% of the

measurements data has been removed based on these principles.

Statistical method

The statistical parameters the normalized mean bias (NMB), and the normalized mean absolute error (NMAE) were used to evaluate the accuracy of the estimation model. These estimators were defined as follows:

$$\mathbf{NMB} = \left(\frac{\sum_{i=1}^{i=N}(M_i - O_i)}{\sum_{i=1}^{i=N}(O_i)}\right) = \left(\frac{\bar{M}}{\bar{O}} - 1\right) \qquad (1)$$

$$\mathbf{NMAE} = \left(\frac{\sum_{i=1}^{i=N}|M_i - O_i|}{\sum_{i=1}^{i=N}(O_i)}\right) = \frac{E_{MAGE}}{\bar{O}} \qquad (2)$$

$$\bar{M} = \frac{1}{N}\sum_{i=1}^{i=n} M_i$$

Where M_i and O_i represented the calculated and measured data, respectively; N is the sample number; and $\bar{O}$ is the average measured data.

The metric of NMB was this parameter can indicate both the magnitude of the factor between the modelled and observed results, and the factor for greater or less than unity. If NMB is positive indicated that the model overestimates the observations by a factor of NMB+1. On the contrary negative NMB represented the model underestimates the observations by a factor of 1−NMB. NMAE can repented the absolute gross error between observation and model prediction results.

Method

The influence of cloud and AOD on *R_s* in Beijing

As mentioned above, the main influence factors for R_s are aerosols, column ozone and clouds. To explore the consistency of R_s attenuation caused by cloud with the cloud value, we used the surface observed cloud cover provided by the Meteorological Information Comprehensive Analysis and Process System (MICAPS) from 2002. In cloudy conditions, the transmittance due to cloud extinction τ_c is a function of sunshine duration [*Ångström*, 1924]. *Yang* et al. [2006] used a two-pass procedure to calibrate the model: select data in pass one, and calibrate the model in pass two. The calibrated cloud-related transmittance for daily solar radiation estimation can be expressed as Eq. (4). In Yang's study, the calibrated model and 3 other models were used to estimate the daily solar radiation at 7 validation stations in China. The calibrated model yielded the least averaged root mean square error. In our study, τ_c was also calculated using Eq. (3).

$$\tau_c = 0.2505 + 1.468\, n/N_s - 0.3974(n/N_s)^2 \qquad (4)$$

where n/N_s represents the relative sunshine duration during a certain period, n is the actual sunshine duration obtained from the CMA routine weather stations, and N_s is the maximum possible sunshine duration, the length of time for which solar direct normal irradiance exceeds a threshold value of 120 $W \cdot m^{-2}$ in clear sky conditions. The units of n and N_s are both hours. The daily surface solar radiation R_s ($MJ \cdot m^{-2} \cdot d^{-1}$) under cloudy skies can be expressed as follows:

$$\boldsymbol{R}_{\mathbf{s}} = \boldsymbol{\tau}_{\mathbf{c}}\boldsymbol{R}_{\mathbf{clear}} \quad (5)$$

where R_{clear} is the daily surface solar radiation under clear skies that can be expressed as follows:

$$R_{clear} = \int_{t\,=\,24h} (\tau_b + \tau_d) R_0 dt \quad \textbf{(6)}$$

$$\boldsymbol{\tau}_{\mathbf{b}} = \boldsymbol{\tau}_{\mathbf{r}}\boldsymbol{\tau}_{\mathbf{a}}\boldsymbol{\tau}_{\mathbf{oz}}\boldsymbol{\tau}_{\mathbf{w}}\boldsymbol{\tau}_{\mathbf{g}} \quad \textbf{(7)}$$

$$\boldsymbol{\tau}_{\mathbf{d}} = \mathbf{0.5}[\boldsymbol{\tau}_{\mathbf{oz}}\boldsymbol{\tau}_{\mathbf{g}}\boldsymbol{\tau}_{\mathbf{w}}(\mathbf{1} - \boldsymbol{\tau}_{\mathbf{a}}\boldsymbol{\tau}_{\mathbf{r}})] \quad \textbf{(8)}$$

where τ_b is the solar beam radiative transmittance; τ_d is the solar diffuse radiative transmittance under clear skies; and $\tau_r, \tau_a, \tau_{oz}, \tau_w$, and τ_g are the transmittance functions of Rayleigh scattering, aerosol extinction, ozone absorption, water vapour absorption and permanent gas absorptionres, respectively.

The contribution of cloud to R_s attenuation can be calculated as follows:

$$b_c = (R_{sc} - R_s) / R_{snof} *100\% \quad \textbf{(9)}$$

where R_{sc} is the solar radiation reaching the Earth's surface without cloud attenuation; R_s is the solar radiation reaching the Earth's surface after attenuation by AOD, ozone, water vapour and cloud; and R_{snof} is the solar radiation reaching the Earth's surface without attenuation by any of these four factors.

Similar to Eq. (9), the contribution of AOD to R_s attenuation can be obtained using the following equation:

$$b_a = (R_{sa} - R_s) / R_{snof} *100\% \quad \textbf{(10)}$$

where R_{sc} is the solar radiation reaching the Earth's surface without aerosol attenuation.

The daily surface solar radiation can be calculated from surface pressure, surface relative humidity, air temperature and sunshine duration obtained from CMA routine weather stations.

Evaluate the performance of the estimation equation for other stations located in NCP

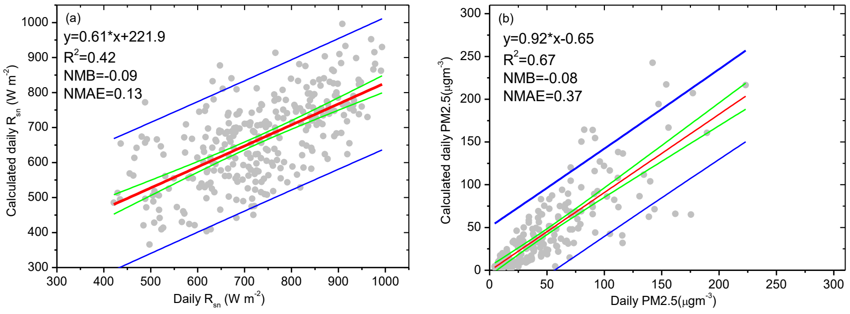

Figure S2. Scatter plot of measured and calculated daily R_{sn} and PM2.5 concentration in Shangdianzi station. (a) Comparison of measured and modelled R_{sn}; and (b) comparison of measured and modelled PM2.5. The fitted regression line (in red), the 90% confidence limits (in blue), and the 95% prediction limits (in green) are displayed. The figure was produced using OriginPro.

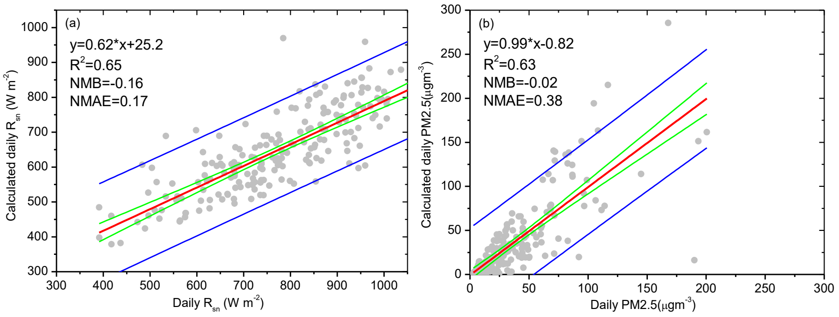

Figure S3. Same as Figure S2 but for Xianghe station. The figure was produced using OriginPro.

To test the transferability of this relationship, developed based on the Beijing station, the performance of the R_{sn} and concentration of PM2.5 estimation model was evaluated using the *in situ* data measured at Shangdianzi and Xianghe stations during 2013-2014 (Fig. S2 and S3). This figure shows that there was a good linear relationship between the measured and modelled values, and approximately 99% calculated data fell within the 90% confidence level limits. The slope of the linear regression was less than 1 and lower than that obtained in Beijing station, which indicated that this method underestimates R_{sn}. The NMB and NMAE were -0.16 and 0.17, respectively. The statistical results of linear regression between calculated and observed concentration of daily average PM2.5 show that the slope was near 1, and the NMB and NMAE were -0.02 and 0.38, respectively. In the same way, the performance of the R_{sn} and concentration of PM2.5 estimation model has been evaluated by using the *in situ* measured data at Xianghe station from April 2013 to December 2014 (Fig. S3a and S3b). The NMB and NMAE for R_{sn} were -0.16 and 0.17, respectively. The NMB and NMAE for PM2.5 concentration were -0.02 and 0.38, respectively. When we compared these results to the relative error between the measured and inversely calculated PM2.5 concentration and R_{sn} in Beijing, this method provides an acceptable

calculated PM2.5 concentration and R_{sn}. However, there is a significant underestimation of R_{sn} at locations other than Beijing, suggesting that this method should be modified according to *in situ* measured results.

Daily variation characteristic of R_{sn} and PM2.5 concentration in Beijing

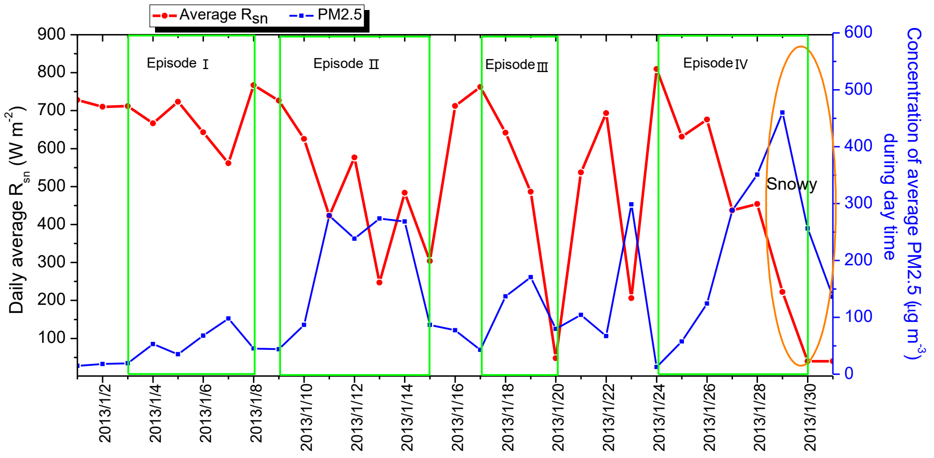

Figure S4 Periodic R_{sn} and PM2.5 cycles in Beijing during the heavy haze pollution episode of January 2013. The figure was produced using OriginPro.

The *in situ* measured data from the January 2013 heavy haze pollution episode has been used to investigate variation trends of R_{sn} and the concentrations of $PM_{2.5}$ in Beijing. From Figure S4, we can find there is a significant opposite change trend when comparing R_{sn} with the concentration of $PM_{2.5}$.

Ångström (1929, 1930) proposed that the extinction of spherical particles depends on wavelength and this can be expressed as a simple power law equation, viz.

$$AOD(\lambda) = \beta \times \lambda^{-\alpha} \quad (11)$$

Where λ is the wavelength, β is the turbidity coefficient and α is the Ångström exponent.

For a given wavelength λ_{ref}, the aerosol optical depth can be expressed as:

$$AOD(\lambda_{ref}) = \beta \times \lambda_{ref}{}^{-\alpha} \quad (12)$$

***AOD*(*λ*) is obtained by *AOD*(λ_{ref}):**

$$AOD(\lambda) = AOD(\lambda_{ref}) \times (\lambda / \lambda_{ref})^{-\alpha} \quad (13)$$

Similarly, *AAOD*(*λ*) can be obtained by *AAOD*(λ_{ref}) using the following equation:

$$AAOD(\lambda) = AAOD(\lambda_{ref}) \times (\lambda / \lambda_{ref})^{-AAE} \quad (14)$$

Where *AAE* is the absorption Ångström exponent.

Absorbed aerosol is mainly composed of dust, BC and organic carbon aerosols (OC). Thus, $AAOD(\lambda)$ is the sum of the dust absorption optical depth ($AAOD_{dust}(\lambda)$), BC absorption optical depth ($AAOD_{BC}(\lambda)$) and OC absorption optical depth ($AAOD_{OC}(\lambda)$).

$$AAOD(\lambda) = AAOD_{dust}(\lambda_{ref}) \times (\lambda / \lambda_{ref})^{-AAE_{dust}} + AAOD_{BC}(\lambda_{ref}) \times (\lambda / \lambda ref)^{-AAE_{BC}} + AAOD_{OC}(\lambda_{ref}) \times (\lambda / \lambda_{ref})^{-AAE_{OC}} \quad (15)$$

Where AAE_{dust}, AAE_{BC} and AAE_{OC} is the dust absorption Ångström exponent, BC absorption Ångström exponent and OC absorption Ångström exponent respectively.

Chen (2012) studied *AAE* and the scatter Ångström exponent (*SAE*) of 35 AERONET stations in East Asia and concluded the *AAE* values for dust, BC and OC (Table S1). The absorption of OC at 870 nm is close to zero and $AAOD_{OC}(870)$ is assumed to zero.

Table S1 The *AAE* values of dust, BC and OC in EAST Asia

Aerosol species	*AAE* (440-675 nm)	*AAE* (675-870 nm)
dust	2.24	1.29
BC	0.33	0.69
OC	4.21	/

Combined the *AAE* values in Table 1 and *AAOD*(440), *AAOD*(675) and *AAOD*(870) from Beijing station of AERONET, $AAOD_{dust}(675)$, $AAOD_{BC}(675)$ and $AAOD_{OC}(675)$ can be solved from Eq.(14). The single scattering albedo (SSA) and asymmetry parameter (ASY) for BC is 0.19 and 0.64 from Chung's study (2012), who conducted a sensitivity experiment of SSA and ASY. When SSA changed from 0.19 to 0.3, the surface radiation forcing increased only 1.62% and when ASY changed from 0.64 to 0.55, the surface radiation forcing increased 2.27%.

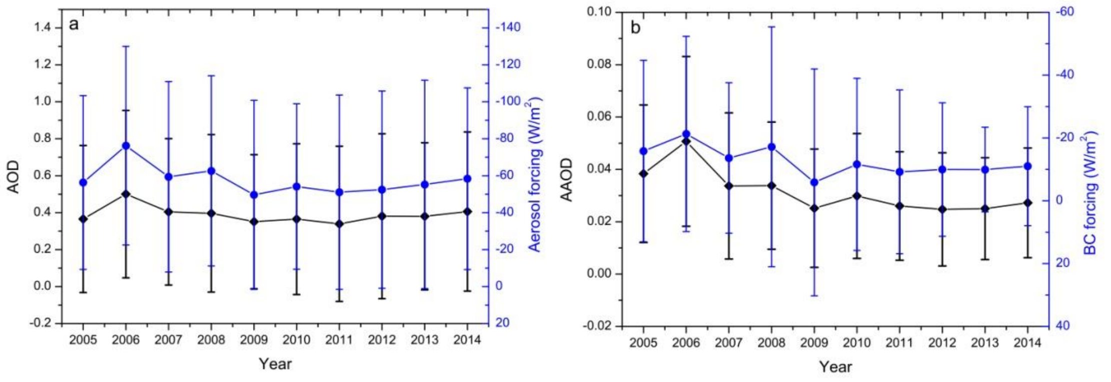

Figure S5. Time series of the AOD and AAOD vs the DRF at surface caused by aerosol and BC during 2005-2015 (a) the variation of AOD and the impact of

DRF caused by AOD, (b) the variation of AAOD and the impact of DRF caused by BC. The figure was produced using OriginPro.

The surface radiation forcing of composite aerosol and BC from 2005 to 2014 in clear sky conditions is showed in Figure S5 (b). A significantly decreasing trend from 2006 to 2009 is presented which is in agreement with PM2.5 and opposite with R_s. After 2010, no significant variation trend appeared for aerosol and BC radiation forcing. The forcing values were 57.55 W m^{-2} and 12.56 W m^{-2} for composite aerosol and BC respectively.

References

Ångström, A., (1924), Solar and terrestrial radiation. Report to the international commission for solar research on actinometric investigations of solar and atmospheric radiation. *Q. J. Roy. Meteor. Soc.*, 50(210), 121–126.

Angstrom, A., (1929), On the atmospheric transmission of Sun radiation and on the dust in the air. Geografis. Ann., 11, 156 - 166.

Angstrom, A., (1930), On the atmospheric transmission of Sun radiation. Geografis. Ann., 12, 130 - 159.

Bruce, W. (1996), A new method for calibrating reference and field pyranometers, J. Atmos. Ocean Tech., 13, 638– 645.

Chen B., (2012), Detection Light-Absorbing Aerosols and their Properties from Satellite and AERONET observations over East Asia. Lanzhou University.

Chung C.E., V. Ramanathan, D. Decremer., (2012), Observationally constrained estimates of carbonaceous aerosol radiative effect Proc Natl Acad Sci USA, 109, 11624–11629

Geiger, M., L. M. Diabate, and L. Wald (2002), A web service for controlling the quality of measurements of global radiation, Solar Energ., 73, 475–480.

Determination of atmospheric articles PM10 and PM2.5 in ambient air by gravimetric method HJ 618-2011. Ministry of Environment protection of the people's republic of China.

Song Guo, Min Hu, Misti L. Zamora et al.(2014), Elucidating severe urban haze formation in China, Proc. Natl. Acad. Sci. USA 111, 17373

Yang, K., T. Koike, and B. S. Ye (2006), Improving estimation of hourly, daily, and monthly solar radiation by importing global data sets, *Agr. Forest. Meteorol.*, *137*(1-2), 43-55, doi:10.1016/j.agrformet.2006.02.001.

Acknowledgments

We thank the U.S. Geological Survey for providing the topographic information for drawn figure S1 (ftp://edcftp. cr.usgs. gov/ pub/ data/ gtopo30/ global from http://edc.usgs.gov).
